# CD34 Stem Cell Boost in Pediatric Allogeneic Stem Cell Transplant Recipients: A Case Series and Review of Literature

**DOI:** 10.1007/s44228-023-00042-w

**Published:** 2023-04-07

**Authors:** Sara Bowman, Joe Stanek, Rajinder Bajwa, Veronika Polishchuk, Rolla Abu-Arja, Hemalatha G. Rangarajan

**Affiliations:** 1 Department of Pediatric Hematology, Oncology, Blood and Marrow Transplant, Nationwide Children’s Hospital, 700 Children’s Drive, Columbus, OH USA; 2 Department of Pediatrics, The Ohio State University, Columbus, OH USA; 3 Biostatistics Resource at Nationwide Children’s Hospital, Columbus, OH USA

**Keywords:** CD34 stem cell boost, Outcomes, Post stem cell transplant, Pediatrics

## Abstract

Patients with poor graft function (PGF) or declining donor chimerism (DC) post allogeneic hematopoietic cell transplantation (HCT) may benefit from a CD34-selected stem cell boost (SCB). We retrospectively studied outcomes of fourteen pediatric patients (PGF: 12 and declining DC: 2), with a median age of 12.8 (range 0.08–20.6) years at HCT, who received a SCB. Primary and secondary endpoints included resolution of PGF or improvement in DC (≥ 15% increase), overall survival (OS) and transplant-related mortality (TRM), respectively. The median CD34 dose infused was 7.47 × 10^6^/kg (range 3.51 × 10^6^–3.39 × 10^7^/kg). Among patients with PGF who survived ≥ 3 months post-SCB (*n* = 8), we observed a non-significant decrease in the cumulative median number of red cell transfusions, platelet transfusions, and GCSF but not intravenous immunoglobulin doses in the 3 months before and after SCB. Overall response rate (ORR) was 50%, with 29% complete and 21% partial responses. ORR was better in recipients who received lymphodepletion (LD) pre-SCB versus none (75% versus 40%; *p* = 0.56). The incidence of acute and chronic graft-versus-host-disease was 7% and 14%, respectively. The 1-year OS was 50% (95% CI 23–72%) and TRM was 29% (95% CI 8–58%). SCB was effective in half of our cohort with possible benefit of LD pre-SCB.

## Introduction

Allogeneic hematopoietic stem cell transplantation (HCT), a curative option for many hematological malignancies and disorders, can be associated with poor graft function (PGF) and mixed chimerism (MC). Depending on the underlying primary diagnosis, PGF/MC may necessitate further interventions such as a rapid taper or increase in immunosuppression, stem cell boost (SCB), donor lymphocyte infusion (DLI), or a second HCT [1]. PGF is defined as frequent dependence on blood and/or platelet transfusions and/or growth factors in the absence of other explanations such as disease relapse, drugs or infection [1]. PGF occurs in 5–27% of patients after initial HCT and is associated with a high mortality rate, most often secondary to infection [2]. Mixed chimerism is defined as donor chimerism (DC) between 5 and 95% [3] for both myeloid and lymphoid lineages [1], and occurs with greater frequency following transplant for non-malignant diseases (NMD) and with the use of reduced intensity (RIC) or non-myeloablative conditioning regimens [4].

While PGF can be managed with blood product transfusions or growth factors [Granulocyte colony stimulating factor (G-CSF), thrombopoietin agonists or erythropoietin] [5], this may not be sustainable in the long-term, and is associated with side effects such as iron overload, alloimmunization, thrombosis and bone marrow fibrosis. Similarly, declining DC portends risk of relapse/recurrence of the underlying disease. When compared to DLI, a CD34 SCB is associated with decreased risk of graft versus host disease (GvHD) and, hence, may be the preferred intervention to improve GF or DC [6, 7]. A ^second^ HCT, on the other hand, has an increased risk of transplant-related mortality (TRM) and morbidity [8, 9]. While the current literature mostly focuses on outcomes of SCB post-HCT for PGF in adults [2, 3, 10–12], there have only been a few pediatric studies that have analyzed outcomes post-SCB in select diseases, such as immunodeficiencies [3] or malignancies [13–15]. Therefore, we retrospectively evaluated the efficacy and outcomes of pediatric patients who received a CD34 SCB post-HCT at our center. Two patients in this series have been previously reported [16, 17]. We also reviewed the literature on outcomes of this intervention in pediatric HCT recipients.

## Methods

### Patients

We obtained approval from our institution’s research board for this retrospective study. All patients who received a CD34 SCB from their original HCT donor for either PGF or MC at our center, from January 2014 to December 2021, were included. Data on demographics, HCT, SCB characteristics, and clinical outcomes were collected and entered into a secure database. PGF was defined as cytopenia(s) affecting any hematopoietic cell line(s) (ANC < 1000/μl, Hb < 8 g/dL, platelets < 30,0000 K/µL) for at least 2 weeks post-HCT requiring the support of transfusions or growth factors in the presence of full DC and absence of relapse, severe GvHD, viral reactivation, and/or drug-related myelosuppression [15]. MC was defined as DC between 5 and 95% in either whole, myeloid, or lymphoid lineages, whereas full DC was defined as having ≥ 95% in all lineages [3]. Stem cells were obtained by apheresis in all but one patient who received a bone marrow (BM) boost. For the former, donors received 10 µg/kg of GCSF for 5 days prior to apheresis for peripheral blood stem cells. The donor apheresis product underwent CD34 selection using the CliniMACS® Milteny device and the fresh product was then infused in patients [18].

### Response Criteria

The primary endpoint was improvement in PGF or MC. Responses for PGF were categorized as complete response (CR: resolution of all cytopenias), partial response (PR: resolution of some, but not all cytopenias), or no response (NR) based on recovery of the underlying parameter at least 30 days post-SCB [10]. Improvement in MC was defined as a rise in DC by ≥ 15% and having DC ≥ 20% 3 months post-SCB; stabilization was defined as a rise in DC by < 15% but with DC ≥ 20% 3 months after the boost.^1^ NR was defined as a decline/no change in DC at 3 and 12 months post-SCB or the need for a second allogenic HCT [3]. Secondary endpoints included cumulative incidence of acute and chronic GvHD, 1-year overall survival (OS), and TRM. OS was defined as the time from SCB to time of death from any cause or censoring. TRM was defined as deaths without signs of relapse of primary disease post-SCB. Acute and chronic GvHD were graded according to Glucksberg’s criteria [19] and NIH consensus guidelines, respectively [20].

### Statistical Analysis

Descriptive statistics were used to summarize all the data. Frequencies and percentages were used to summarize categorical variables, and median and range for quantitative variables. Wilcoxon signed-rank tests were used to compare the number of pRBC and platelet transfusions, GCSF, and IVIG doses before and after SCB. The comparison of CR + PR i.e., overall response rate (ORR) among those who did and did not receive lymphodepletion (LD) was done with a Fisher’s exact test. OS was estimated using the Kaplan–Meier method and presented with a corresponding 95% confidence interval (CI). The TRM rate was summarized as a percentage and 95% CI. *P* values less than 0.05 were considered statistically significant. We compared the median cumulative number of pRBC, platelet transfusions, GCSF and IVIG doses in the three-month preceding and following SCB in patients with PGF who survived ≥ 3 months post-SCB. Analyses were completed using SAS software, version 9.4 (SAS Institute, Cary, NC).

## Results

**Fig. 1:**
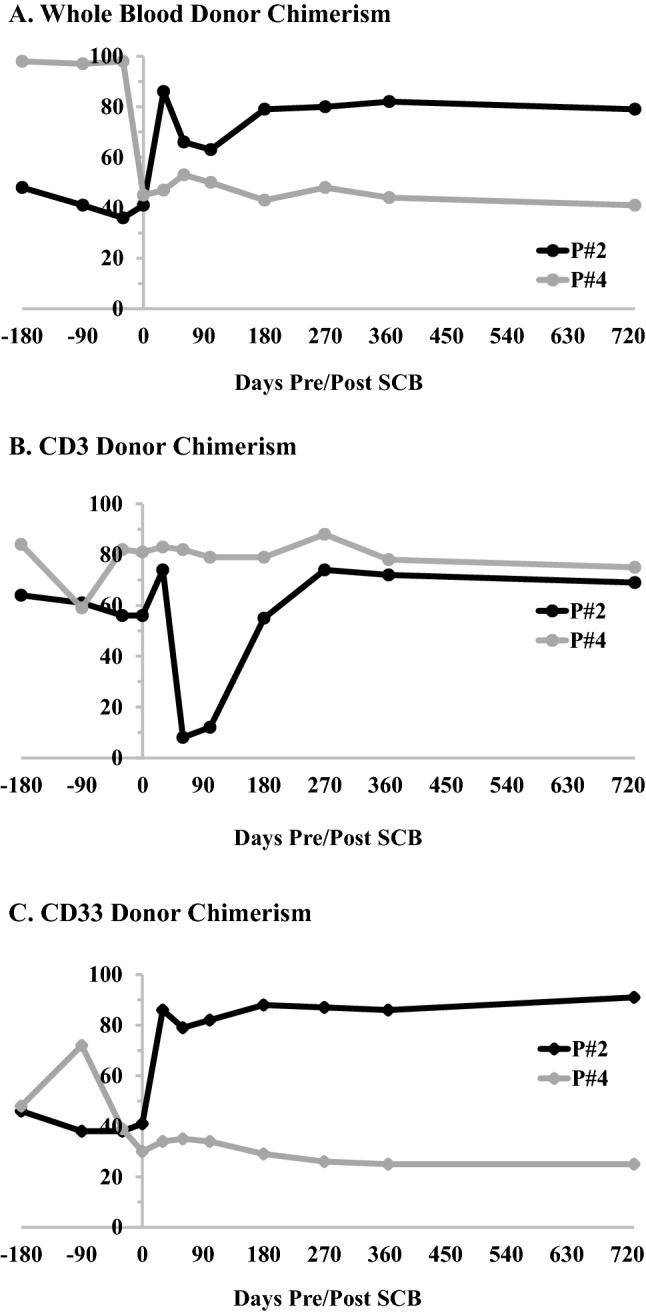
Donor chimerism in 2 patients pre and post SCB **A** whole blood **B** CD3 **C** CD33 fractions

Fourteen (7 male) allogeneic HCT recipients, with a median age of 12.8 (range 0.08–20.6) years at HCT, received a CD34 SCB during the study period (Table 1). This included 5 patients with a malignant disease and 9 with a NMD. Nine received myeloablative (MA) and 5 RIC regimens. Amongst these, one patient (P#11) received a 2nd MA HCT following graft failure (GF) post-1st HCT. Bone marrow (BM; *n* = 12) or peripheral blood stem cells (PBSC; *n* = 2) were obtained from matched sibling donors (MSD; *n* = 3), matched unrelated donors (MUD; *n* = 7), and haploidentical donors (*n* = 4). Twelve (86%) had PGF and 2 (14%) had declining DC pre-SCB (Fig. 1). Prior to SCB, all but two patients had a history of infections with five (42%) patients with PGF undergoing active treatment for infections at the time of SCB (Table 2). BM biopsy performed in 10 patients with PGF pre-SCB demonstrated a median cellularity of 15% (range 0–60%). Amongst patients with PGF, 1 had single-line cytopenia (P#1: thrombocytopenia), 1 had bi-lineage cytopenia (P#6: anemia, neutropenia), and 8 had pancytopenia. Thrombopoietin agonists agonists were not considered for the P#1 due to risk BM fibrosis in a heavily treated patient with therapy-related AML/MDS. All patients received blood products (pRBCs, platelets) and growth factors ± intravenous immunoglobulin (IVIG) for ongoing cytopenias. P#11 (Severe congenital neutropenia) and P #12 (OMS, Omenn’s syndrome) developed B-cell aplasia and required monthly IVIG for approximately 4 and 19 years, respectively, pre-SCB. While P#11 developed B cell aplasia following rituximab therapy for EBV reactivation, P#12 had B cell aplasia in the setting of persistently low donor CD19 chimerism (3%) with recurrent episodes of bacterial sinusitis. In the two patients with MC, DC pre-SCB were: whole blood (WB) 41 and 45%; CD3: 56 and 81%; CD33: 30 and 41%, respectively. Despite rapid weaning of immunosuppression (tacrolimus) over 2 weeks, there was no improvement in DC in either of these patients. P#2 was also subsequently treated with a 3-month course of sirolimus with no effect. Six patients developed acute GvHD (Grade I-II in 4, Grade III-IV in 2 patients), and one patient developed severe chronic GVHD following their HCT, all of which resolved prior to a SCB [10].

**Table 1:** HCT and SCB characteristics of study cohort (*n* = 14)

Pt	Pt demo	HCT demo	D/R CMV status	SCB indication	HCT-SCB interval	SCB characteristics	Post-SCB Immunosuppression and duration (months) New Onset GvHD (if applicable)	Outcomes
	Age (yr), sex, diagnosis	Donor Type Source, Prep				Conditioning (if applicable) CD34 dose (× 10^6^/kg) CD3 dose (× 10^3^/kg)		Responses in months post SCB: CR/PR/NR Survival post-SCB Last F/u from SCB, Cause of Death
1	10.7, F, AML	MUD BM, MAC	±	PGF Thrombocytopenia	1 yr	6.55; 9.35	Jakafi, Steroids*	CR @ 1mo; Died @ 10mo; Relapse
2	13.9, F, SCD	MUD BM, RIC	±	MC	1 yr, 9mo	Flu, CTX, ATG 6.95; 6.46	Tacrolimus, 3mo	CR @ 9mo; Alive @ 25mo
3	15.1, M, AML	MUD BM, MAC	±	PGF Pancytopenia	3mo	4.96; 1.51	Tacrolimus, 9mo Chronic GvHD – mild	CR @ 2mo; Died @ 41mo; Relapse
4	1.8, M, ICF	MSD BM, RIC	±	MC	6mo	6.43; 1.77	–	NR; Alive @ 45mo
5	17.2, F, HL	MSD PBSC, RIC	−/+	PGF Pancytopenia	2mo	3.51; 0.72	Tacrolimus*	PR @ 1mo; Died @ 1mo; Progressive Disease
6	11.7, F, CVID	MUD BM, MAC	−/+	PGF Anemia, Neutropenia	1 yr, 1mo	Flu, TBI, Alemtuzumab 7.88; 1.61	–	PR @ 1mo; Died @ 2mo; RV Failure secondary to anemia
7	13.9, M, AutoImm	MUD BM, RIC	–/–	PGF Pancytopenia	7mo	7.25; 1.48	Tacrolimus, Steroids, MMF*	NR; Died @ 1mo; Disseminated Fungal Infection
8	17.1, F, AML	MUD BM, MAC	+/+	PGF Pancytopenia	1mo	9.98; 1.01	–	NR; Died @ 1mo; Hepatorenal Failure
9	20.6, M, ALL	Haplo PBSC, MAC	−/+	PGF Pancytopenia	2mo	7.69; 0.78	Tacrolimus, 2mo	PR @ 0.5mo; Died @ 3mo; Refractory Disease
10	2.8, M, MDS	Haplo BM, MAC	±	PGF Pancytopenia	2mo	TBI, Flu, CTX, ATG 33.9; 6.93	Tacrolimus, 15mo Chronic GvHD—mild	CR @ 2mo; Alive @ 36mo
11	0.5; 1.5, F, SCN	MUD BM, MAC	±	PGF B-cell Aplasia	4 yr, 2mo	14.2; 1.47	–	NR; Alive @ 39mo
12	0.1, F, SCID (OMS)	Haplo BM, MAC	±	PGF B-cell Aplasia	18 yr, 11mo	7.93; 1.87	–	NR; Alive @ 13mo
13	4.8, M,^#^ SCD	MSD BM, MAC	–/–	PGF Pancytopenia	3mo	Flu, ATG 6.53; 3.10 × 10^7^	Cyclosporine, 6mo	NR; Alive @ 108mo Had secondary GF and return of SCD
14	14.5, M, VSAA	Haplo BM, RIC	+/+	PGF Pancytopenia	9mo	11.0; 3.39	Tacrolimus, Steroids* Acute GvHD GradeP#14 II	NR; Died @ 6mo: Disseminated Fungal + Bacterial Infection

Pt Patient, Demo Demographics, M Male, F Female, AML Acute Myeloid Leukemia, SCD sickle cell disease (Hgb SS), ICF Immunodeficiency centromeric region instability facial anomalies syndrome, HL Hodgkin’s Lymphoma, CVID Common Variable Immunodeficiency, AutoImm Autoimmune Disorder, ALL Acute Lymphoblastic Leukemia, MDS Myelodysplastic Syndrome, SCN Severe Congenital Neutropenia, SCID Severe Combined Immunodeficiency, OMS Omenn Syndrome, VSAA Very Severe Aplastic Anemia, MSD Matched sibling donor, MUD Matched unrelated donor, Haplo haploidentical, MAC Myeloablative chemotherapy, RIC Reduced-Intensity chemotherapy, BM Bone Marrow, PBSC Peripheral blood stem cells, D/R: Donor/Recipient CMV: cytomegalovirus PGF Poor graft function, MC Mixed chimerism, Flu Fludarabine, CTX Cyclophosphamide, ATG Anti-thymocyte globulin, TBI total body irradiation, CR Complete Response, PR Partial Response, NR No Response

*Remained on Immunosuppression until date of death

^#^Patient received a BM boost

**Table 2:** HCT and SCB-related infections in study cohort (*n* = 14)

Pt	Infections pre-SCB	Status of infection at time of SCB	Infections post-SCB
1	Viral reactivations: BK, Adenovirus, EBV, CMV Treated with: Cidofovir, Adenoviral CTLS, Rituximab and Ganciclovir *All resolved 11 months pre-SCB*	No active infection	None
2	Infections: C.diff Treated with: Metronidazole	No active infection	Infections: EBV (resolved 1-month post SCB) Treated with: Rituximab
3	Viral reactivations: CMV and HHV6 Treated with: Ganciclovir (IV), then Valganciclovir (oral); IVIG	Active: CMV reactivation Treatment: Valganciclovir	Viral Reactivations: CMV Treated with: Valganciclovir (resolved 9 months post-SCB)
4	Infections: Adenoviremia Treated with: Self-resolved	No active infection	None
5	Infections: Adenoviremia, CMV Treated with: Cidofovir, CTLs *Resolved one month prior to SCB*	No active infection	None
6	Infections: Adenoviremia Treated with: Cidofovir (IV)	No active infection	Infections: Adenoviremia Treated with: Cidofovir (resolved one-month post-SCB)
7	Infections: Fusarium, Adenovirus, BK Viremia Treated with: Micafungin, Amphotericin, Brincidofovir	Active: Fusarium, BK Viremia Treatment: Micafungin, Amphotericin, Brincidofovir	Infections: Fusarium, BK Viremia Treated with: Micafungin, Amphotericin, Brincidofovir (treated until death)
8	Infections: CMV viremia, Candidemia Treated with: Foscarnet, Amphotericin	Active: CMV viremia, Candidemia Treatment: Foscarnet, Amphotericin	Infections: CMV viremia, Candidemia Treated with: Foscarnet, Amphotericin (treated until death)
9	Infections: BK viremia, Nocardia Treated with: Cidofovir, Bactrim, Ceftriaxone, Linezolid	Active: BK Viremia Treatment: Cidofovir	Infections: BK Viremia, Nocardia Treated with: Cidofovir, Bactrim, Ceftriaxone (treated until death)
10	Infections: Norovirus diarrhea Treated with: Notaxonazinde *Resolved one-month pre-SCB*	No active infection	None
11	None	No active infection	None
12	Infections: Recurrent bacterial sinusitis Treated with: Antibiotics	No active infection	None
13	None	No active infection	Infections: EBV viremia Treated with: Rituximab
14	Infections: CMV, BK and parvovirus Treated with: Letermovir, Cidofovir, BK CTLS, High dose IVIG	Active: CMV, BK and parvovirus Treatment: Letermovir, Cidofovir, High dose IVIG	Infections: CMV, BK, parvovirus, disseminated *Candida glabrata and Enterococcus faescium* Treated with: Letermovir, Cidofovir, High dose IVIG, Antibiotics

HCT Hematopoietic stem cell transplant, SCB Stem cell boost, Pt Patient, EBV Ebstein-Barr virus, CMV Cytomegalovirus, CTL Cytotoxic T-Lymphocytes, C.diff Clostridium difficile, HHV6 Human herpesvirus 6

The median interval between SCB and prior HCT was 0.43 years (range 0.1–19.2). Three patients with PGF (P # 6, 11, and 12) received a CD34 SCB more than 1-year post- HCT. P#6 had autoimmune hemolytic anemia (AIHA) with neutropenia that was refractory to treatment with several agents (steroids, cyclosporine, eculizumab, and daratumumab). Therefore, she received LD followed by a CD34 SCB 13 months post-HCT. P#11 and P#12 were treated for prolonged B cell aplasia as previously described.

**Fig. 2:**
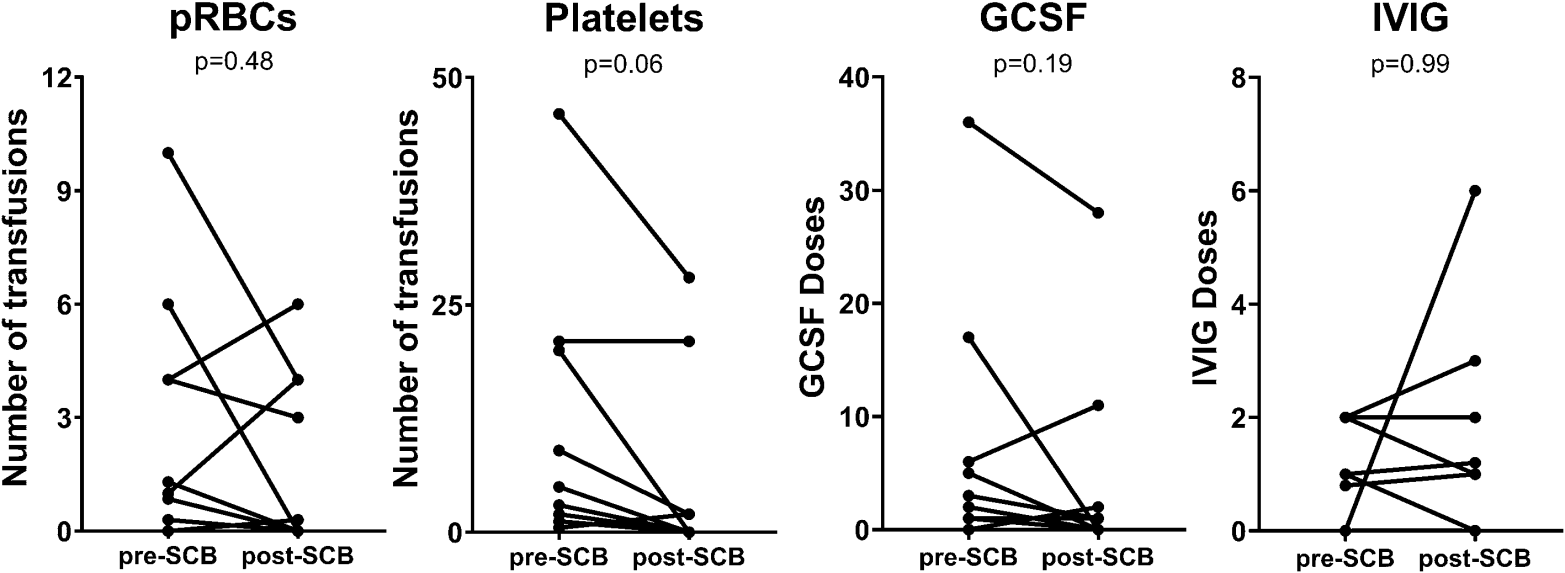
Number of packed red blood cell and platelet transfusions, GCSF and IVIG doses in the 3 months pre and post SCB in patients with PGF (*n* = 8)

Only one patient in our cohort received a marrow boost (P#13). The median CD34 and CD3 doses infused were 7.47 × 10^6^/kg (range 3.51 × 10^6^–3.39 × 10^7^/kg) and 1.74 × 10^3^/kg (range 7.2 × 10^2^–3.10 × 10^7^/kg). Nine patients were briefly maintained on immunosuppression post-SCB (Table 1). Amongst the 12 patients with PGF, after excluding four who died < 3 months post-SCB, in the remaining eight there was a non-significant decrease in the median cumulative number of pRBC [pre: 1 (range 0–6) versus 0 (range 0–6); *p* = 0.48], platelet transfusion [pre: 4 (range 0–21) versus 0 (range 0–21); *p* = 0.06], GCSF [pre: 2 (range 0–36) versus post: 0 (range 0–28); *p* = 0.19], but not IVIG doses [pre: 1 (range 0–2) versus 1 (range 0–6); *p* = 0.99] (Fig. 2).

The ORR in our cohort at last follow-up was 50% (*n* = 7), with CR in 29% (*n* = 4) and PR in 21% (*n* = 3). Responses were observed at a median of 1-month post-SCB. The ORR in those with MC was 50% (1 CR, 1 NR) **(**Fig. 1**)** and 50% in those with PGF (3 CR, 3 PR, 6 NR). Amongst the latter, P#1 with single-line cytopenia had a CR, P#6 with bi-lineage cytopenia had a PR, and of the 8 with pancytopenia, there were 2 CR, 2 PR, and 4 NR. The two patients with B-cell aplasia had NR and continued to receive monthly IVIG at the last follow-up. Comparing those with malignant diagnosis versus NMD, 50% of those with history of a malignancy had a CR (*n* = 3), 33% a PR (*n* = 2), and 17% NR (*n* = 1); 12.5% of those with NMD had a CR (*n* = 1), 12.5% had a PR (*n* = 1), and 75% had NR (*n* = 6). P#13, who received a BM boost, had NR and subsequently developed GF with return of sickle cell disease. Four patients (P#2, 6, 10, and 13) in our cohort received LD chemotherapy pre-SCB. This included 1 patient with MC (P#2) and 3 with PGF (Table 1). The ORR in patients who received LD (*n* = 4) and those who did not (*n* = 10), was 75% (2 CR, 1 PR, 1 NR) versus 40% (2 CR, 2 PR and 6 NR), respectively (*p* = 0.56).

**Fig. 3:**
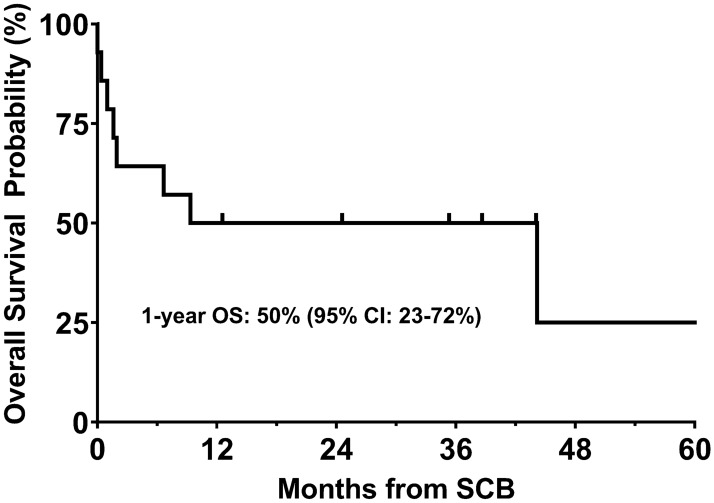
Overall survival of Study Cohort

Following SCB, new infections occurred in 4 patients and these included viral reactivations: EBV (P#2 and 13), adenovirus (P#6) and disseminated *Candidemia* and *Enterococcus faescium* (P#14) **(**Table 2). New onset GVHD post-SCB occurred in three patients: Grade II aGVHD in one (7%) and mild cGVHD in 2 patients (14%). At a median follow up of 1.96 years (range 0.0025–9.05 years) post-SCB, 43% (*n* = 6) of patients were alive. The 1-year OS was 50% (95% CI 23–72%) and TRM was 29% (95% CI 8–58%) (Fig. 3). Causes of mortality included relapse of malignancy (*n* = 4), disseminated fungal infection (*n* = 2), right heart failure secondary to anemia (*n* = 1), and hepatorenal failure (*n* = 1).

## Discussion

A CD34-selected SCB, unlike DLI, is associated with decreased risk of GvHD and, hence, may be a preferred treatment strategy for PGF or MC following an allogeneic HCT [6, 7]. Prabahran and colleagues described three mechanisms causing PGF: a decline in the number or efficacy of the stem cells (seed), abnormalities in the BM microenvironment (soil), and immunosuppression of hematopoiesis (environment) [21]. The discussed potential treatment options were CD34-selected SCB, TPO agonists and G-CSF (seed), mesenchymal stromal cells, and antioxidants (soil), T regulatory cell infusion, emapalumab and JAK inhibition (environment). A CD34 SCB is particularly advantageous in patients with NMDs, where graft-versus-leukemia and associated GvHD are of no benefit. However, if there is no response to a SCB by 3 months, an alternative treatment should be pursued. [3]. Through our report, we further demonstrate the effectiveness of a SCB in pediatric HCT recipients. We observed an ORR of 50% in our study cohort including 29% CR and 21% PR. We also noted a decrease in requirements for blood products and GCSF doses in the 3 months post-SCB in patients with PGF. There was, however, no decrease in IVIG requirements in patients with prolonged B cell aplasia. One of the two patients with MC had a CR. A greater proportion of patients who received LD pre-SCB had a response than those who did not.

Three pediatric and three mixed pediatric/adult studies have reported on the use of a SCB for PGF or MC in pediatric patients (Table 3) [12]. While two of these reports [3, 10] focus only on patients with primary immunodeficiencies (PID), the remainder, like ours, included patients with both malignant and NMD [2, 11, 12, 22]. Parallel with our observations, the OS, aGvHD, and cGvHD rates in these studies were 39–100%, 0–12%, and 0–6.2% (Table 3**)**. In contrast, unmanipulated DLI, was associated with 40–60% risk of aGvHD (20–35% with grade III–IV aGvHD) and 33–61% risk of cGvHD, resulting in a decreased OS.[7, 23] In a recent large metanalysis of 209 adults who received a SCB for PGF, the overall pooled ORR, CR and PR were 80%, 72%, and 13%, respectively, but that study did not delineate the type of cytopenia pre-SCB. [6] While the CR rate in our study (29%) is lower than that and those from a few pediatric studies (50–79%) [2, 10, 12], it is similar to that reported by Mianaridi (36%) [11] and Chandra (25%) [3] **(**Table 3). Per the latter’s report, only 1/3 of patients with MC respond to a SCB. Therefore, the high incidence of pancytopenia (80%) amongst patients with PGF and the inclusion of two patients with MC could be a potential explanation for the lower CR rate in our study.

**TABLE 3:** Review of pediatric patients who have received a CD34 selected stem cell boost as reported in literature

Study	No. patients (age range years)	Primary diagnoses		Indication for SCB	Response rates	Overall survival (OS)	Acute and chronic GVHD rates
		Malignant	Non-malignant				
Slatter [10]	19 (N/A)	0	19 [SCID (9), CD40L (3), CGD (2), ICF, XLP, ZAP70, WAS, CHH]	PGF (19)	63% CR 16% PR 21% NR	48% @ 24 months	Acute 10% Chronic 0%
Mainardi [11]	50 (11–87)	41 [ALL (23), AML (11), Solid tumor (3), MDS, CML, NHL, CMML]	9 [SAA (5), Osteopetrosis (2), Thalassemia, WAS]	PGF (50)	36% CR 42% PR 22% NR	39% @ 60 months	Acute 6% Chronic 0%
Chandra [3]	12 (0–20)	8 [HLH]	4 [CGD (2), CID, IFNG-2]	MC (12)	25% CR 8% PR 67% NR	100% @ 32 months	Acute 0% Chronic 0%
Cuadrado [12]	62 (10–66)	59 [LPD (30), AML (11), ALL (7), MDS (7), SAA (3), MF (2), CML]	3 [PID (2), SCD]	PGF (62) MC (32)	63% CR 13% PR 24% NR	53% @ 60 months	Acute 11.3%, Chronic 8%
Berger [2]	16 (0–18)	11 [ALL (5), NHL (3), MPS (2), AML]	5 [SAA (2), DKC, SCD, Fanconi]	PGF (13) MC (3)	50% CR 31% PR 19% NR	56% @ 10 years	Acute 12.5%, Chronic 6.25%
Fraint [22]	14 (0–24)	7 [ALL (4), AML, CML, MDS]	7 [SAA (5), CGD, WAS]	PGF (14)	79% CR 0% PR 14% NR	78% @ 60 months	Acute 7%, Chronic 0%
Our study	14 (0–20)	5 [AML (3), ALL, HL]	9 [SCD (2), ICF Type 1, CI, AD, MDS, CN, SCID, SAA]	PGF (12) MC (2)	29% CR 21% PR 50% NR	57% @ 24 months	Acute 7%, Chronic 21%

SCID Severe Combined Immunodeficiency, CD40L CD40 Ligand Deficiency, CGD Chronic Granulomatous Disease, ICF Immunodeficiency Centromeric Instability Facial Dysmorphism Syndrome, XLP X-Linked Lymphoproliferative Disease, ZAP70 ZAP70 Combined Immunodeficiency, WAS Wiskott-Aldrich Syndrome, CHH Cartilage Hair Hypoplasia, ALL Acute Lymphoblastic Leukemia, AML Acute Myeloblastic Leukemia, SAA Severe Aplastic Anemia, MDS Myelodysplastic Syndrome, CML Chronic Myelogenous Leukemia, NHL Non-Hodgkins Lymphoma, CMML Chronic Myelomonocytic Leukemia, HLH Hemophagocytic Lymphohistiocytosis, CID Combined Immunodeficiency, IFNG-2 IFN-gamma receptor 2, LPD Lymphoproliferative Disorder, MF Myelofibrosis, PID Primary Immunodeficiency, SCD Sickle Cell Disease, HL Hodgkin's Lymphoma, AD Autoimmune Disorder, CN Congenital Neutropenia, SCID Severe Combined Immunodeficiency, CR Complete response, PR Partial response, NR No response

All patients in our cohort received fresh products from their donors. In two studies involving pediatric patients, cryopreserved products were administered to half of the patients [11, 22]. Ghobadi et al. [24] showed that a CD34 SCB from a cryopreserved product, though associated with a lower CD34 yield, still had equally effective responses in treated patients (63% CR) when compared to those who got fresh products (61% CR). In a study of PID patients who received a SCB for PGF or MC, two patients who received a BM boost, achieved CR but then developed GVHD [10]. The singular patient who received a BM boost in our study had NR and did not develop GVHD. A pediatric study involving 16 patients found that a higher proportion of recipients who received a CD34 cell dose > 6.6 × 10^6^/kg (78%) had a response compared to those who received < 6.6 × 10^6^/kg (57%) [2]. A study with 50 patients, however, found no difference in CD34 dose between responders and non-responders. They observed a threshold effect at 3.25 × 10^6^ /kg, which resulted in an optimal increase in neutrophil count, with higher doses showing no further increases [11]. All patients in our study received a dose greater than 3.25 × 10^6^/kg, and all but two patients (1CR and 1PR) received a dose greater than 6.5 × 10^6^/kg.

Multiple studies have demonstrated that response rates are higher for single-lineage compared to bi-lineage or tri-lineage cytopenias. For instance, Berger et al. reported response rates of 80% for single-lineage and 44% for bi-lineage cytopenia in their patients [2, 22]. Other factors predictive of a response to SCB include absence of infection, recipient-donor gender matching, shared donor/recipient (D/R) CMV seronegative status, absence of CMV reactivation [12] and donor age < 40 years [11]. Donor type (sibling versus non sibling) did not seem to impact outcomes in most studies [1, 11]. We could not determine the impacts of these factors in our limited cohort, as all but 2 (1 CR and 1 PR) patients with PGF had pancytopenia. Additionally, only 2 D/R pairs shared CMV seronegative status with NR in both recipients (Table 1). We also found a similar rate of response in gender mismatched cohorts (2 CR 1 PR and 3 NR) versus gender matched (2 CR, 2 PR, 4NR) cohorts (data not shown).

Similar to the Fraint et al.’s report, nearly half of our patients with PGF had active infections around the time of the SCB [22]. Infections not only cause PGF but can also be a major cause of failure of response and deaths following SCB [2]. In our limited cohort, amongst the 7 with NR, 3 had active infections at the time of SCB resulting in death in 2 of these patients (Table 2). Patients with a CR following a SCB have been shown to have a better OS compared to those with a PR or NR [11, 12, 25]. The 5-year OS in a mixed adult-pediatric study of 62 patients was 74%, 17% and 22% in patients with a CR, PR and NR, respectively [12]. In contrast, we did not detect such a difference, which may be attributed to our small sample size. In our study, 50% of patients with CR, 0% with PR, and 58% with NR were alive at the last follow-up. Notably four patients with NR had NMD with two of them receiving a SCB > 1 year post HCT. Therefore, these factors likely contributed to their favorable OS at last follow-up. Berger et al. found that the majority of deaths occurred within the first 6 months of a SCB [2], consistent with our observation wherein six of the eight deaths occurred within the same timeframe.

Like our study, Mainardi et al. also noted a significant decrease in red cell and platelet transfusions (1 and 7 versus 0) at 8-weeks post-SCB from non-sibling donors [11]. Slatter et al. reported improved B cell function and IVIG independence in 7 of 12 responders, and a CR in a patient with AIHA. However, Fraint et al. reported death in a patient with AIHA, despite an initial CR [22]. In our cohort, the two patients with B cell aplasia had NR, and the single patient with AIHA had a PR that was not sustained. Berger et al. excluded patients who received chemotherapy/LD, prior to SCB. However, we elected to include these patients as LD is often used to eliminate residual host immunity thought to mediate PGF/MC, thereby facilitating engraftment [16]. We utilized this strategy in patients with NMD or MDS who are usually chemotherapy-naïve with a robust host immune response, which can mediate graft rejection. Slatter et al. used LD [anti-thymocyte globulin (ATG) or alemtuzumab] pre-SCB in two patients with PID following which one had a CR, the other NR and proceeded to a 2nd HCT. Fraint et al. also included 4 patients with MC (DC < 90%) and pancytopenia due to PGF who received LD (3 with ATG and 1 with Fludarabine and ATG). The outcomes of these patients were not reported separately. [22] Our study was limited by a small sample size and its retrospective nature; despite this, our observations mirror other published reports and add to the literature on the utility of SCB for treatment of PGF or MC after HCT for both malignant and NMD.

## Conclusion

SCB was associated with lower rates of GvHD in our cohort and a non-significant but decreased need for blood product support and GCSF in patients with PGF. Patients with NMD/MDS may benefit from lymphodepletion prior to SCB. Overall, our data support the use and consideration of SCB as a strategy for treatment of PGF or MC after initial HCT in pediatric patients.

## Data Availability

These will be made available upon reasonable request to authors.

## Abbreviations

HCT: Hematopoietic cell transplant
PGF: Poor graft function
MC: Mixed chimerism
DC: Donor chimerism
GvHD: Graft versus host disease
SCB: Stem cell boost
OS: Overall survival
ORR: Overall response rate
CR: Complete response
PR: Partial response
NR: No response
DLI: Donor lymphocyte infusion
NMD: Non-malignant diseases
LD: Lymphodepletion
aGVHD: Acute graft versus host disease
RIC: Reduced intensity chemotherapy
MAC: Myeloablative chemotherapy
NRM: Non-relapse mortality
TRM: Transplant related mortality
BM: Bone marrow
PBSC: Peripheral blood stem cells
MSD: Matched sibling donor
MURD: Matched unrelated donor
SCN: Severe congenital neutropenia
OMS: Omenn syndrome
IVIG: Intravenous immunoglobulin
WB: Whole blood
MDS: Myelodysplastic syndrome
GCSF: Granulocyte colony stimulating factor
cGVHD: Chronic graft versus host disease
